# Lifestyle Score and Risk of Hypertension in the Airwave Health Monitoring Study of British Police Force Employees

**DOI:** 10.3390/ijerph20054029

**Published:** 2023-02-24

**Authors:** Ghadeer S. Aljuraiban, Rachel Gibson, Doris S. M. Chan, Paul Elliott, Queenie Chan, Linda M. Oude Griep

**Affiliations:** 1 Department of Community Health Sciences, College of Applied Medical Sciences, King Saud University, Riyadh 11451, Saudi Arabia; 2 Department of Nutritional Sciences, King’s College London, London SE1 9NH, UK; 3 Department of Epidemiology and Biostatistics, School of Public Health, Imperial College London, London W2 1PG, UK; 4 MRC Centre for Environment and Health, School of Public Health, Department of Epidemiology and Biostatistics and the NIHR Imperial Biomedical Research Centre, Imperial College London, London SW7 2AZ, UK; 5 MRC Epidemiology Unit, University of Cambridge, Cambridge CB2 0QQ, UK

**Keywords:** hypertension, blood pressure, cardiovascular risk, healthy lifestyle

## Abstract

Background: Evidence suggest that promoting a combination of healthy lifestyle behaviors instead of exclusively focusing on a single behavior may have a greater impact on blood pressure (BP). We aimed to evaluate lifestyle factors and their impact on the risk of hypertension and BP. Methods: We analyzed cross-sectional health-screening data from the Airwave Health Monitoring Study of 40,462 British police force staff. A basic lifestyle-score including waist-circumference, smoking and serum total cholesterol was calculated, with a greater value indicating a better lifestyle. Individual/combined scores of other lifestyle factors (sleep duration, physical activity, alcohol intake, and diet quality) were also developed. Results: A 1-point higher basic lifestyle-score was associated with a lower systolic BP (SBP; −2.05 mmHg, 95%CI: −2.15, −1.95); diastolic BP (DBP; −1.98 mmHg, 95%CI: −2.05, −1.91) and was inversely associated with risk of hypertension. Combined scores of other factors showed attenuated but significant associations with the addition of sleep, physical activity, and diet quality to the basic lifestyle-score; however, alcohol intake did not further attenuate results. Conclusions: Modifiable intermediary factors have a stronger contribution to BP, namely, waist-circumference and cholesterol levels and factors that may directly influence them, such as diet, physical activity and sleep. Observed findings suggest that alcohol is a confounder in the BP–lifestyle score relation.

## 1. Introduction

Cardiovascular disease (CVD) is a leading cause of death worldwide, with cardiovascular incidents accounting for almost 85% of total CVD mortality [1]. Hypertension, or high blood pressure (BP), is a major risk factor for cardiovascular morbidity [2] identified as the greatest single preventable cause of mortality worldwide [3].

Hypertension is highly influenced by well-established behavioral lifestyle risk factors, such as smoking and unhealthy diets, and other intermediary factors such as hyperlipidemia and central adiposity [4]. Promoting a healthy lifestyle is an effective approach for improving high BP; however, most studies supporting hypertension prevention recommendations assessed only the single effects of, for example, physical activity (PA) [5] or other lifestyle factors [6], and only a few investigated lifestyle factors concurrently, adding weight to the concept that multiple factors can exert a greater effect when considered together [7,8,9,10]. However, most available scoring systems, such as the QRISK1 and QRISK2 scores [11], used in the National Institute for Health and Care Excellence (NICE) guidelines (including obesity, smoking, serum cholesterol, and other factors [12]) and the American Heart Association’s Life’s Simple 7 (comprising seven modifiable behavioral factors—smoking, body mass index (BMI), PA, diet, cholesterol, BP, and fasting blood glucose [13,14]) have been established to reduce the risk of CVD. Whether these scores apply to the risk of hypertension is yet to be investigated. Further, risk factors included in previous studies were limited to either young adults or only a few risk factors at a time, thus not capturing the multitude of other lifestyle factors that may lower the risk of hypertension further, e.g., sleep [5,15,16,17,18,19,20,21].

Therefore, combining lifestyle factors instead of exclusively focusing on each may significantly impact BP [22] and can be more far-reaching since individual lifestyle recommendations showed differential effects in specific subgroups [23]. In light of this, to promote targeted interventions and identify which lifestyle factors have greater impact on BP/hypertension, the current study aimed to evaluate: a basic lifestyle-score (including available factors from the QRISK2 score [11]); individual lifestyle factors and their combined scores; and the inclusion of individual lifestyle factors to the basic-score. Cross-sectional data from the Airwave Health Monitoring Study, the first large cohort investigating the health of the police workforce in Great Britain [24], comprising a major resource for biomedical research with 42,112 enrolled by the end of 2012 [24] were used. Uniquely, this cohort allows for the consideration of job strain and working patterns specific to the police force, which could impact the achievement and maintenance of a healthy lifestyle [24], and will help in evaluating healthy lifestyle-factors in a population faced with unique occupational challenges.

## 2. Materials and Methods

### 2.1. Study Design

The study design and recruitment details have been published previously [24]. In brief, the study launched in 2004 and a total of 53,114 members of the police force were enrolled by end of 2015. All participants provided written informed consent, and the study ethics were approved by the National Health Service Multi-Site Research Ethics Committee (MREC/13/NW/0588). For this analysis, participants who attended health-screening measurements between 2007 and 2015 were included. Those diagnosed with diabetes or CVD and those with missing data of key variables required for this analysis, e.g., BP, PA, sleep duration, waist-circumference, smoking, and biochemical data were excluded (*n* = 12,652). The final sample included (*n* = 40,462) adults (25,382 men and 15,080 women).

### 2.2. Clinic Visit

Participants were invited for health screening at study clinics, where, following a standard protocol, trained staff conducted clinical examinations and average measurements were used in the analyses. Non-fasting venous blood samples were collected on-site and transported to the study-laboratory to assess levels of serum total and HDL cholesterol (IL650-analyser Instrumentation Laboratory, Bedford, MA, USA). All laboratory equipment were quality assured and controlled. Weight and height were measured twice with participants wearing light clothes, without shoes or socks using a Marsden H226 portable stadiometer and weighing scale. Waist-circumference was measured twice between the lower rib and the iliac crest in the mid-axillary line using a Wessex-finger/joint measure tape. BP was measured three times, 30 s apart, after participants were seated and relaxed (Omron HEM 705-CP, OMRON Corp., Kyoto, Japan). Hypertension was defined as having a systolic BP (SBP) ≥ 140 mmHg and a diastolic BP (DBP) ≥ 90 mmHg [25], or self-reported diagnosis or the intake of anti-hypertensive medication.

### 2.3. Socio-Demographic and Lifestyle Data

Participants completed a self-administrated electronic questionnaire providing socio-demographic and lifestyle data (e.g., age, sex, and education-level). Job strain was measured using the Karasek Job Content Questionnaire [26] which uses the quadrant approach [27] to categorize participants under high (low control, high demand), active/passive (high control, high demand)/(low control, low demand), and low strain (high control, low demand). Physical activity (PA) was assessed using the short version of the International PA Questionnaire [28]. The questionnaire asks participants to report the frequency and duration of domain-specific activities and energy expenditure in metabolic equivalent minutes/week, and based on this data, intensity of activities (high, moderate, or low) are assigned [28].

### 2.4. Dietary Data

A subsample of participants (*n* = 8546) completed 7-day food diaries to report their dietary intake. Photographs and common household measures developed by Nelson et al. were provided [29] for better portion-size estimation. Details on cooking methods and brand names were included. For quality-control, trained nutritionists/dietitians followed a study-specific operational manual to code the diaries and match food/drink items recorded to a UK Nutritional database code and a portion-size [30]. For nutrient-analysis, Dietplan software (version 6.7; Forestfield Software Ltd., Horsham, UK) based on the UK nutrient-database of McCance and Widdowson [31] was used.

### 2.5. Nutrient-Rich Food 9.3 Index-Score

Diet-quality was assessed using the Nutrient-Rich Food 9.3 (NRF9.3) index-score [32], reported to be highly correlated with the Healthy Eating Index, a measure of diet quality-score established by the US Dietary Guidelines [33]. For the NRF9.3 index-score calculation, the sum of the percentage of daily nutrient values of nine nutrients to encourage (protein, dietary fiber, vitamins A, C, E, calcium, iron, potassium, and magnesium) minus the sum percentage of maximum recommended values for three nutrients to restrict (saturated fat, added sugar, and sodium) per 100 kcal was computed. A higher NRF9.3 index-score reflects higher-nutrient quality per 100 kcal.

### 2.6. Lifestyle-Score 

A basic lifestyle-score including available factors from the QRISK2 score [11]; waist-circumference, smoking and serum cholesterol (Table 1) was calculated. For the basic lifestyle-score, participants were stratified into three mutually exclusive categories: poor (0–3 points), intermediate (4 points), and ideal (5–6 points).

Additionally, other individual lifestyle factors likely to be on the causal pathway for the risk of hypertension (sleep duration, PA, alcohol intake, and diet quality) and their combined scores were calculated. Participants were also stratified into three mutually exclusive categories: poor, intermediate, and ideal. Each lifestyle factor was defined as poor, intermediate, and ideal, following the 2020 Impact Goals definitions [14]. Ethnic/gender-specific cut-offs for waist-circumference [12,34] were used. For PA, the American Heart Association guide for assessing PA was applied [35]. For sleep, the American Academy of Sleep Medicine and Sleep Research Society [36] guidelines were applied to identify poor (≤5 or ≥9 h), intermediate (6 h), and ideal (7–8 h) amounts of sleep. For diet quality, participants were classified based on published cut-offs of a similar UK sample population [37] into poor (NRF9.3 < 15), intermediate (NRF9.3 16–25), and ideal (NRF9.3 > 25) diet quality.

To evaluate the impact of these lifestyle factors on the basic lifestyle-score relative to BP/hypertension, additional scores were calculated by adding one factor at a time to the basic lifestyle risk-score, defined as follows: a basic lifestyle-score + sleep duration, a basic lifestyle-score + sleep duration + PA, a basic lifestyle-score + sleep duration + PA + alcohol intake, and a basic lifestyle-score + sleep duration + PA + alcohol intake + diet quality (in a subsample *n* = 8546).

### 2.7. Statistical Analysis

To calculate scores, ideal levels were given 2 points, intermediate 1 point, and poor 0 points. The sum of points for each lifestyle factor was used to calculate the cumulative score, with the lowest possible score being zero (poor levels of all factors) and the highest for all seven factors being 14 (ideal levels of all factors).

Baseline characteristics of participants were presented according to levels of the basic lifestyle-score (ideal (5–6 points), moderate (4 points), and low (0–3 points)) using a linear age, sex, and employment country-adjusted model to assess the linearity of the investigated relations.

Associations of lifestyle factors with BP were evaluated using multivariate linear-regression models adjusted for age, sex, and employment country. Subsequently, two sequential multivariate linear regression models adjusted for potential confounders were used to determine associations with BP for each 1-point higher basic lifestyle-score. Further, individual lifestyle factors and their combined scores were investigated in relation to BP. Finally, the relative impact of each lifestyle factor on the basic lifestyle-score was assessed by adding one factor at a time to the basic lifestyle-score. Logistic regression analysis was applied to estimate the odds of hypertension per total and levels of the lifestyle-scores. Stratified analyses and interaction terms were applied, detecting no evidence of the potential effect modification by age, sex, and BMI. Despite no evidence of effect modification, and given that the average age of participants was relatively young (mean = 40.4 (SD = 8.9) y), participants were stratified by age (≤30, 30 to ≤40, 40 to ≤50, >50 y) and the linear regression analysis was repeated to gain more insight into the relation with BP.

To investigate whether the main findings were independent of characteristics such as self-reported diagnosis of hypertension, antihypertensive drug use, and prevalent major chronic diseases (e.g., diabetes), the multivariate linear regression analyses were repeated in a sub-cohort of participants with characteristics that might bias the association between the basic lifestyle-score and BP. A sub-cohort of participants was identified with a self-reported diagnosis of hypertension and users of antihypertensive drugs and with prevalent cardiovascular diseases and diabetes mellitus from the foregoing cohort (*n* = 5686). Additionally, a sub-cohort excluding energy mis-reporters from 8546 participants who completed the dietary data was defined using the Goldberg equation (*n* = 7567) [38]. The SAS version 9.3 (SAS Institute, Cary, NC, USA) was used to perform the statistical analysis; *p* values < 0.05 were considered statistically significant.

## 3. Results

### 3.1. Demographic and Lifestyle Characteristics of the Sample

The sample included 40,462 participants with an average age (mean (SD)) of 40.5 (8.9) years. Overall, 95% of the participants were White and 63% were men (Table 2). When participants were stratified by the basic lifestyle-score, about 30% had poor, 26% intermediate, and 44% ideal lifestyle-score.

### 3.2. Association between the Basic Lifestyle-Score and BP/Hypertension

A 1-point higher basic lifestyle-score was associated with SBP/DBP differences of −2.05/−1.98 mmHg (Model 2; Table 3). 

**Figure 1:**
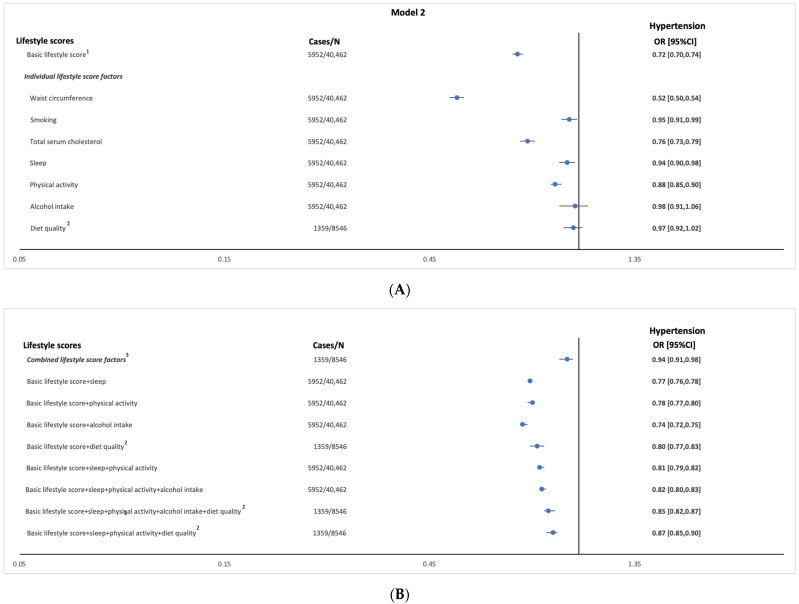
Odds ratio [95%CI confidence intervals] of hypertension per 1-point increase in total (**A**) basic, individual, and (**B**) combined lifestyle score factors in a sample of the Airwave Health Monitoring Study (*n* = 40,462). Model 1 is adjusted for age, sex, and employment country. Model 2 is model 1 adjusted for marital status, education, ethnicity, annual household income, and history of chronic diseases. Hypertension was defined as having SBP ≥ 140 mmHg or DBP ≥ 90 mmHg, reported diagnosis or on anti-hypertensive medication ^1^ Basic lifestyle score includes (waist-circumference + smoking + cholesterol) ^2^ Analyzed in a subsample of *n* = 8546 ^3^. Combined lifestyle score factors include (sleep + physical activity + alcohol intake + diet quality).

Logistic regression analyses showed a significant relationship between the basic lifestyle-score and the odds of hypertension (OR per 1 point increase = 0.72 (95%CI: 0.70, 0.74)) (Model 2, Figure 1A and Table S1).

**Figure 2:**
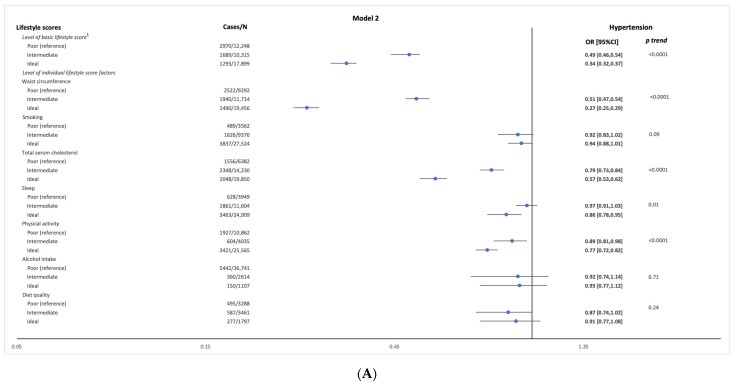

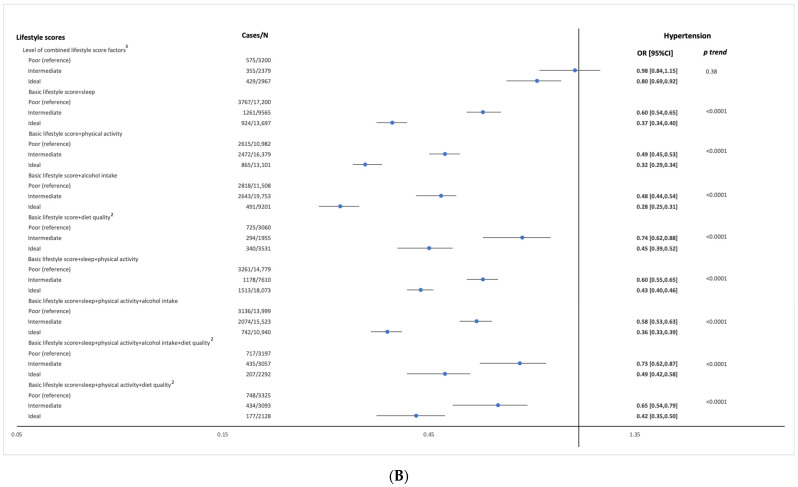
Odds ratio [95%CI confidence intervals] of hypertension per 1-point increase in levels of (**A**) basic, individual, and (**B**) combined lifestyle score factors in a sample of the Airwave Health Monitoring Study (*n* = 40,462). Model 1 is adjusted for age, sex, and employment country. Model 2 is model 1 adjusted for marital status, education, ethnicity, annual household income, and history of chronic diseases. Hypertension was defined as having SBP ≥ 140 mmHg or DBP ≥ 90 mmHg, reported diagnosis or on anti-hypertensive medication ^1^ Basic lifestyle score includes (waist-circumference + smoking + cholesterol) ^2^ Analyzed in a subsample of *n* = 8546 ^3^. Combined lifestyle score factors include (sleep + physical activity + alcohol intake + diet quality).

Across levels of the basic lifestyle-score, the odds of having hypertension decreased with scoring higher for the basic lifestyle-score, with ORs being 0.49 (95% CI: 0.46, 0.54) for intermediate level and 0.34 (95% CI: 0.32, 0.37) for the ideal level compared with the poor level (Model 2, Figure 2A and Table S1). Age-stratified multivariate regression analysis showed that the association between the basic lifestyle-score and BP was stronger in the older age groups (40 to ≤50 and >50 years), (SBP: −2.46 (95%CI: −2.62, −2.29); DBP: −2.25 (95%CI: −2.36, −2.14)) and (SBP: −2.34 (95%CI: −2.70, −1.98); DBP: −1.72 (95%CI: −1.93, −1.52)) compared to the younger age groups (Table S2).

### 3.3. Association of Individual Lifestyle Factors and Their Combined Scores with BP/Hypertension

A 1-point higher waist-circumference-score was associated with −3.63 mmHg lower SBP (95% CI: −3.80, −3.47) and a −3.53 mmHg lower DBP (95% CI: −3.64, −3.42). Similarly, smoking, cholesterol, sleep duration, PA, alcohol intake, and the NRF9.3 index-score were associated with lower SBP and/or DBP (Model 2; Table 3).

Logistic regression analyses only showed significant associations between waist-circumference, smoking, cholesterol, sleep duration, and PA scores, and the odds of hypertension (Model 2, Figure 1A and Table S1). Across levels of each individual lifestyle factor, the odds of having hypertension decreased with scoring higher for waist-circumference, cholesterol, sleep duration (only for ideal vs. poor level), and PA (Model 2, Figure 2A and Table S1).

When lifestyle-score factors (sleep duration + PA + alcohol intake + diet quality) were combined, the association attenuated with −0.18/−0.62 mmHg lower SBP/DBP (Model 2; Table 3). 

Significant associations were observed between combined lifestyle-score factors and the odds of hypertension (Model 2, Figure 1B and Table S1). Across the levels of combined lifestyle-score factors, the odds of having hypertension decreased with scoring higher for combined lifestyle-score factors with OR being 0.80 (95%CI: 0.69, 0.92) for the ideal level compared with the poor level (Model 2, Figure 2B and Table S1).

Age-stratified analysis showed comparable results of individual score relations to BP, with a trend of stronger associations between waist-circumference-score and BP in older compared to younger participants (Table S2). However, relations between the combined individual scores and BP attenuated and were no longer statistically significant in age-stratified analysis (Table S2). 

### 3.4. Association of Inclusion of Individual Lifestyle Factors to the Basic Score with BP/Hypertension

The relative impact of each lifestyle factor on the basic lifestyle-score showed that the association with SBP and DBP attenuated when adding sleep duration, PA and diet quality, but remained statistically significant (Model 2, Table 3). However, the addition of alcohol intake to the basic lifestyle-score only slightly altered the results (SBP −1.91 (95% CI: −2.00, −1.81; DBP −1.85 (95% CI: −1.92, −1.79)) mmHg.

When alcohol was added to the basic lifestyle-score + sleep + PA, it did not further attenuate the results (SBP −1.07 (95% CI: −1.14, −1.00; DBP −1.27 (95% CI: −1.32, −1.22)) mmHg (Model 2, Table 3). Thus, in model 3, sleep + PA + diet quality was added to the basic model and adjusted for alcohol intake; however, the results remained the same. 

The relationship with the odds of hypertension also attenuated but remained significant when all other lifestyle components (sleep duration, PA, alcohol intake, and diet quality) were added to the basic lifestyle-score (Model 2, Figure 1A and Table S1). Associations prevailed across the levels of lifestyle-score factors included in the basic score (Model 2, Figure 2A and Table S1).

For age-stratified analysis, lifestyle factors included in the basic lifestyle-score showed a stronger trend in the relation with BP among older (>50 y) compared to younger adults (≤30 y) (Table S2). 

### 3.5. Association of Basic Lifestyle-Score with BP in Sub-Cohorts 

The regression analyses were repeated using model 2 in the sub-cohorts that excluded the participants with characteristics that might bias the associations with BP (e.g., self-reported diagnosis of hypertension, antihypertensive drug use) (Table S3), and found that the results prevailed and remained statistically significant. 

## 4. Discussion

The present large cohort study evaluated cross-sectional associations of lifestyle-scores in relation to BP/hypertension, reporting a 2.0 mmHg lower SBP (an epidemiologically significant difference at the population level [39]) and a 30% lower risk of hypertension for each 1-point higher adherence to a basic lifestyle-score (including waist-circumference, smoking and serum cholesterol). When lifestyle factors were considered individually, only waist-circumference, low serum cholesterol level, and low alcohol intake contributed to a lower SBP and/or DBP and the risk of hypertension, which can be explained by a healthy waist-circumference and low serum cholesterol. Although significance of the associations prevailed, associations attenuated with the addition of sleep duration, PA, and diet quality. Although evaluated in a smaller subsample, a lifestyle-score including sleep duration, PA and diet quality did not show comparable BP-lowering benefits as the basic lifestyle-score. Significantly lower BP was observed with healthier lifestyle-scores in young adults (≤30 y), with a larger mean difference in BP in the older age group (>50 y) compared to younger age groups. 

The relationships between lifestyle factors and BP found here are not surprising given that they were chosen a priori based on the existing literature demonstrating their relationship with BP [4]. It is likely that some lifestyle variables have a stronger contribution to lowering BP than others, namely, more objective ones including waist-circumference and cholesterol levels. Furthermore, when alcohol intake was added to the basic lifestyle-score, it did not further attenuate the results, suggesting that alcohol is a confounder in the relationship, given its relationship with both BP (the outcome) [40], and waist-circumference [41], smoking [42] and serum cholesterol [43] (the exposures). On the other hand, when other factors such as PA, diet, sleep duration, and smoking were added to the basic lifestyle-score, the association with BP attenuated, suggesting that these factors may act as mediators in the association of the basic lifestyle-score with BP. The relationship between these factors and cholesterol or waist-circumference has been well-established [44,45,46,47,48,49]. For example, the attenuation observed when diet and PA were added to the basic lifestyle-score may be attributed to their significant and direct impact on weight and serum cholesterol levels. This suggests that interventions focused on healthier diets and increased PA are important and have the potential to reduce BP and the risk of hypertension [44,50]. Even in young adults, <30 y, lifestyle-scores were related to lower BP, supporting findings that maintaining healthy behaviors from an early age can have favorable impacts on BP and a reduction in hypertension risk [51]. 

The scores evaluated as part of this work demonstrated a significant relationship with the odds of hypertension. Furthermore, the scores are also suggestive of the magnitude of risk with a more ideal lifestyle being associated with a lower risk of hypertension than an intermediate lifestyle, thus, demonstrating the potential value of the score for assessing hypertension risk. Importantly, although the addition of sleep, PA, and diet attenuated the association of the basic lifestyle-score with SBP/hypertension, the lifestyle-score including only sleep, PA and diet (although in smaller subsample) did not show a lower BP/hypertension comparable to the basic lifestyle-score. This suggests that the basic-score cannot be merely replaced by the lifestyle-score including sleep, PA, and diet in this population. 

The present study fills a gap in evaluating the combined impact of several lifestyle factors on BP/hypertension and uses several validated measures for assessing lifestyle data including the International PA Questionnaire [28] and the NRF9.3 [32]. The study used cross-sectional data and therefore a temporal relationship between hypertension and lifestyle factors cannot be established. As with any interview-based data collection, some variables used in the lifestyle-score were subject to misreporting or recall bias. Another consideration is that the Airwave Health Monitoring Study recruits from a distinctive population—those working in the police force [24]. As such, it provides a novel opportunity to study a population with unique occupational challenges. However, the generalizability of the research conducted in this cohort may be limited with the study population being predominantly male with a small proportion of staff from ethnic minorities. It is unknown how well the results can be generalized to the UK population at-large, nor to populations outside of the UK, although underlying biological pathologies are likely to be similar in other groups. Future work can aim to validate and assess the reliability of this tool in the current and other cohorts.

## 5. Conclusions

Given the pervasiveness of hypertension and its contribution to mortality worldwide [3], identifying which lifestyle behaviors impact hypertension risk the most is valuable. This study highlights the value of objective factors including waist-circumference and cholesterol levels that suggested a stronger contribution to BP than others. The combined impact of lifestyle behaviors suggests that alcohol is a confounder in the BP–lifestyle score relationship, and suggests that factors influencing weight, such as diet and PA, may be important in managing the risk of hypertension. Strategies to adopt healthy behaviors may be useful to lower BP and manage hypertension risk by clinicians, researchers, and members of the public, even in young adulthood.

## Figures and Tables

**Table 1:** Definition of poor, intermediate, and ideal BP lifestyle scores for each factor.

Goal/Factor	Poor	Intermediate	Ideal
Waist-circumference	Male: >102 cm, Female: >88 cm (White); Male: >90 cm, Female: >88 cm (other ethnic groups)	Male: ≥94 to ≤102 cm, Female: ≥80 to ≤88 cm (White); Male: ≥85 to ≤90 cm, Female: ≥80 to ≤88 cm (other ethnic groups)	Male: <94 cm, Female: <80 cm (White); Male: <85 cm, Female: <80 cm (other ethnic groups)
Smoking status	Current	Former < 12 months	Never or quit ≥ 12 months
Total serum cholesterol	≥240 mg/dL	200–239 mg/dL	<200 mg/dL
Additional lifestyle factors			
Sleep duration	5 h or less or 9 h or more	6 h	7 and 8 h
Physical activity	None	Recreational walking 1 to 8 h a week or practicing physical activity and sports activities for 1 to 4 h a week	Recreational walking for 8 h or more a week or practicing physical activity and sports activities for more than 4 h a week
Alcohol intake	Current	Former < 12 months	Never or quit ≥ 12 months
Diet quality (nutrient-rich food index 9.3) score	≤16	16–25	>25

**Table 2:** Baseline characteristics stratified by poor, intermediate, and ideal basic lifestyle scores in 40,462 participants of the Airwave Health Monitoring Study ^a,b^.

	Poor (0–3)	Intermediate (4)	Ideal (5–6)	Total
*n*	12,248 (30%)	10,315 (26%)	17,899 (44%)	40,462
Median basic lifestyle score	3	4	5	4
Male (%)	64.3	64.1	62.3	63.3
Age (y)	43.3 (43.2, 43.5)	41.0 (40.9, 41.2)	37.6 (37.5, 37.8)	40.5 (8.9)
Ethnicity (%)				
White	93.1	94.3	96.1	94.8
Marital status (%)				
Cohabiting	14.3	15.7	19.8	17.1
Married	64.8	63.2	56.9	60.9
Divorced/separated	9.5	8.6	7.0	8.1
Single	9.5	10.6	13.6	11.6
Missing	2.0	1.9	2.8	2.3
Education (%)				
Left school before taking GCSE	6.0	3.9	2.2	3.8
GCSE or equivalent	34.7	31.3	25.5	29.8
Vocational qualifications	7.7	7.3	6.4	7.0
A levels/higher or equivalent	29.8	31.6	33.0	31.6
Bachelor’s degree or equivalent	15.9	19.6	25.8	21.2
Postgraduate qualifications	5.9	6.3	7.2	6.6
Annual household income (%)				
Less than £26,000	7.6	7.2	6.4	6.9
£26,000–£37,999	17.4	16.9	17.7	17.4
£38,000–£57,999	37.9	37.7	37.0	37.4
£58,000–£77,999	24.1	24.9	25.6	25.0
More than £78,000	13.0	13.4	13.5	13.3
Employment (force) country (%)				
England	66.9	69.3	71.6	69.6
Scotland	16.7	15.8	15.2	15.8
Wales	14.6	13.0	11.4	12.8
Missing	1.8	1.9	1.8	1.8
Job Strain				
Low (high control, low demand)	23.5	23.2	24.1	23.7
Active/passive (high demand, high control)	49.5	49.8	49.0	49.4
High (high demand, low control) (%)	27.0	27.0	26.9	27.0
Physical activity (%)				
Low	33.7	28.2	21.3	26.8
Moderate	10.0	10.4	9.7	10.0
High	56.3	61.4	69.0	63.2
Smoking status (%)				
Current	21.6	8.6	0.0	8.8
Former (<12 months)	38.5	24.7	11.8	23.2
Never or quit (≥12 months)	39.8	66.7	88.2	68.0
Sleep duration (%)				
5 h or less, 9 h or more	11.4	10.1	8.4	9.8
6 h	31.3	29.8	26.2	28.7
7–8 h	57.3	60.1	65.4	61.5
Alcohol intake (%)				
Current	90.8	91.0	91.0	90.9
Former (<12 months)	6.6	6.4	6.2	6.4
Never or quit (≥12 months)	2.6	2.7	2.8	2.7
Systolic BP (mmHg)	131.9 (131.6, 132.1)	128.8 (128.6, 129.1)	125.6 (125.4, 125.8)	129.9 (15.2)
Diastolic BP (mmHg)	81.9 (81.8, 82.1)	79.3 (79.2, 79.5)	75.9 (75.8, 76.0)	79.2 (10.1)
BMI (kg/m^2^)	29.7 (29.7, 29.8)	27.5 (27.4, 27.6)	24.9 (24.8, 24.9)	27.2 (4.2)
Waist-circumference (cm)	96.2 (96.0, 96.4)	89.7 (89.5, 89.9)	82.0 (81.9, 82.2)	89.8 (12.4)
Total cholesterol (mg/dL)	227.6 (226.9, 228.3)	201.0 (200.3, 201.8)	180.8 (180.2, 181.4)	201.4 (45.2)
Nutrients ^c^				
*n*	3372	3372	1801	
Total energy (kcal)	1977 (1962, 1992)	1820 (1805, 1834)	1693 (1673, 1712)	1895 (480)
Carbohydrates (%)	46 (46, 47)	46 (46, 47)	47 (47, 48)	47 (7)
Protein (%)	16 (15, 16)	17 (16, 17)	19 (19, 20)	17 (3)
Fat (%)	36 (35, 36)	33 (33, 34)	30 (30, 31)	34 (6)
NRF9.3 index score	11.7 (10.8, 11.2)	20.5 (20.3, 20.6)	32.3 (32.1, 32.5)	19.5 (8.5)
NRF9.3 index score components (per 1000 kcal)				
Protein (g)	38 (38, 39)	43 (42, 43)	48 (47, 48)	43 (8)
Fiber (g)	8 (7, 8)	10 (9, 10)	12 (10, 11)	9 (3)
Vitamin A (IU)	1226 (1183, 1268)	1515 (1475, 1556)	2388 (2332, 2443)	1543 (1246)
Vitamin E (mg)	4 (3, 4)	4 (3, 4)	5 (4, 5)	4 (1)
Vitamin C (mg)	32 (30, 32)	49 (48, 50)	81 (80, 82)	48 (29)
Calcium (mg)	434 (430, 438)	464 (460, 468)	498 (493, 503)	452 (109)
Magnesium (mg)	132 (131, 133)	156 (156, 157)	184 (182, 185)	152 (31)
Iron (mg)	6 (5, 6)	6 (5, 6)	7 (6, 7)	6 (2)
Potassium (mg)	1434 (1425, 1443)	1696 (1687, 1704)	2027 (2016, 2039)	1630 (335)
Saturated fatty acid (g)	16 (15, 16)	13 (13, 14)	11 (11, 12)	14 (3)
Total sugar (g)	48 (47, 48)	47 (46, 47)	51 (50, 51)	48 (15)
Total sodium (mg)	1452 (1441, 1464)	1452 (1441, 1462)	1425 (1410, 1440)	1420 (312)

^a^ Mean (95%CI) or (%) ^b^ The generalized linear model was adjusted for age, sex, and employment country ^c^ Analyzed in a subsample of *n* = 8546.

**Table 3:** Estimated mean difference in BP associated with a 1-point higher basic and lifestyle scores and their components in a sample of the Airwave Health Monitoring Study (*n* = 40,462) ^a,b^.

	Systolic Blood Pressure (mmHg)	Diastolic Blood Pressure (mmHg)
	Mean difference (95%CI)	Mean difference (95%CI)
Basic lifestyle score (waist-circumference + smoking + cholesterol)		
Model 1	−2.07 (−2.16, −1.97) ***	−2.01 (−2.08, −1.94) ***
Model 2	−2.05 (−2.15, −1.95) ***	−1.98 (−2.05, −1.91) ***
Model 3	−2.05 (−2.15, −1.95) ***	−1.98 (−2.05, −1.92) ***
Individual lifestyle risk factors		
Waist-circumference		
Model 1	−3.57 (−3.73, −3.40) ***	−3.55 (−3.66, −3.43) ***
Model 2	−3.63 (−3.80, −3.47) ***	−3.53 (−3.64, −3.42) ***
Smoking		
Model 1	−0.14 (−0.35, 0.06)	−0.24 (−0.38, −0.10) **
Model 2	−0.07 (−0.27, 0.14)	−0.23 (−0.38, −0.09) **
Total serum cholesterol		
Model 1	−2.66 (−2.84, −2.47) ***	−2.42 (−2.56, −2.29) ***
Model 2	−2.59 (−2.78, −2.40) ***	−2.36 (−2.49, −2.23) ***
Sleep		
Model 1	−0.05 (−0.25, 0.15)	−0.38 (−0.52, −0.25) ***
Model 2	−0.06 (−0.26, 0.14)	−0.39 (−0.52, −0.25) ***
Physical activity		
Model 1	−0.12 (−0.27, 0.04)	−0.94 (−1.05, −0.84) ***
Model 2	−0.12 (−0.27, 0.04)	−0.94 (−1.05, −0.84) ***
Alcohol intake		
Model 1	−0.76 (−1.09, −0.43) ***	−0.67 (−0.90, −0.44) ***
Model 2	−0.57 (−0.91, −0.24) **	−0.60 (−0.83, −0.36) ***
Diet quality		
Model 1	−0.13 (−0.71, −0.02) *	−0.53 (−0.71, −0.35) ***
Model 2	−0.02 (−0.29, 0.24)	−0.51 (−0.70, −0.33) ***
Combined lifestyle score (sleep + physical activity + alcohol intake + diet quality) ^c^		
Model 1	−0.19 (−0.37, −0.02) *	−0.63 (−0.75, −0.51) ***
Model 2	−0.18 (−0.36, −0.01) *	−0.62 (−0.74, −0.51) ***
Stepwise inclusion of individual lifestyle factors to the basic score		
Basic lifestyle score + sleep		
Model 1	−1.57 (−1.66, −1.49) ***	−1.60 (−1.66, −1.54) ***
Model 2	−1.57 (−1.66, −1.48) ***	−1.58 (−1.64, −1.53) ***
Basic lifestyle score + physical activity		
Model 1	−1.34 (−1.42, −1.26) ***	−1.53 (−1.59, −1.48) ***
Model 2	−1.33 (−1.41, −1.25) ***	−1.52 (−1.57, −1.47) ***
Basic lifestyle score + alcohol intake		
Model 1	−1.95 (−2.05, −1.86) ***	−1.90 (−1.96, −1.83) ***
Model 2	−1.91 (−2.00, −1.81) ***	−1.85 (−1.92, −1.79) ***
Basic lifestyle score + diet quality		
Model 1	−1.41 (−1.59, −1.22) ***	−1.48 (−1.60, −1.36) ***
Model 2	−1.42 (−1.61, −1.24) ***	−1.48 (−1.60, −1.35) ***
Basic lifestyle score + sleep + physical activity		
Model 1	−1.10 (−1.18, −1.03) ***	−1.31 (−1.36, −1.26) ***
Model 2	−1.10 (−1.17, −1.02) ***	−1.39 (−1.44, −1.34) ***
Basic lifestyle score + sleep + physical activity + alcohol intake		
Model 1	−1.10 (−1.17, −1.03) ***	−1.29 (−1.33, −1.24) ***
Model 2	−1.07 (−1.14, −1.00) ***	−1.27 (−1.32, −1.22) ***
Basic lifestyle score + sleep + physical activity + alcohol intake + diet quality ^c^		
Model 1	−0.77 (−0.90, −0.65) ***	−1.00 (−1.05, −0.88) ***
Model 2	−0.78 (−0.90, −0.65) ***	−1.00 (−1.05, −0.88) ***
Basic lifestyle score + sleep + physical activity + diet quality		
Model 1	−0.91 (−1.05, −0.77) ***	−1.11 (−1.21, −1.02) ***
Model 2	−0.92 (−1.06, −0.77) ***	−1.11 (−1.21, −1.02) ***
Model 3	−0.91 (−1.05, −0.77) ***	−1.11 (−1.21, −1.02) ***

^a^ Values are presented as mean (95%CI, confidence intervals); * *p*-value <0.05, ** *p*-value <0.001, *** *p*-value < 0.0001 ^b^ Model 1 is adjusted for age, sex, and employment country. Model 2 is model 1 adjusted for marital status, education, ethnicity, annual household income, and history of chronic diseases. Model 3 is model 2 adjusted for alcohol intake. ^c^ Analyzed in a subsample of *n* = 8546.

## Data Availability

Data are available upon request.

## Supplementary Materials

The following supporting information can be downloaded at: https://www.mdpi.com/article/10.3390/ijerph20054029/s1, Table S1: Odds ratio of hypertension for the basic and lifestyle scores in a sample of the Airwave Health Monitoring Study (*n* = 40,462); Table S2: Estimated mean differences in BP associated with a 1-point higher lifestyle scores and their components in a sample of the Airwave Health Monitoring Study (*n* = 40,462) stratified by age; Table S3: Estimated mean differences in BP associated with a 1-point higher lifestyle scores in sub-cohorts of the Airwave health monitoring study.
