# Supplementary material for: Lifestyle Score and Risk of Hypertension in the Airwave Health Monitoring Study of British Police Force Employees
Source: Int J Environ Res Public Health. 2023 Feb 24;20(5):4029. doi: 10.3390/ijerph20054029 (PMC10001706; doi:10.3390/ijerph20054029)
Supplement: Supplementary file 1 [file ijerph-20-04029-s001.zip › ijerph-2151102-supplementary.pdf]

Table S1. Odds ratio of hypertension for the basic and lifestyle scores in a sample of the Airwave Health Monitoring Study (n=40,462) [a,b]

	Cases/N	Model 1		Model 2		Model 3	
Hypertension[c]		OR	(95% CI)	OR	(95% CI)	OR	(95% CI)
Basic lifestyle score (waist circumference+smoking+cholesterol)	5952/40462	0.72	(0.70, 0.73)	0.72	(0.70, 0.74)	0.72	(0.70, 0.74)
Individual lifestyle score factors							
Waist circumference	5952/40462	0.52	(0.50, 0.54)	0.52	(0.50, 0.54)		
Smoking	5952/40462	0.95	(0.91, 0.99)	0.95	(0.91, 0.99)		
Total serum cholesterol	5952/40462	0.75	(0.72, 0.78)	0.76	(0.73, 0.79)		
Sleep	5952/40462	0.94	(0.90, 0.98)	0.94	(0.90, 0.98)		
Physical activity	5952/40462	0.88	(0.85, 0.90)	0.88	(0.85, 0.90)		
Alcohol intake	5952/40462	0.98	(0.91, 1.05)	0.98	(0.91, 1.06)		
Diet quality [d]	1359/8546	0.98	(0.92, 1.02)	0.97	(0.92, 1.02)		
Combined lifestyle score factors (sleep+physical activity+alcohol intake+diet quality)[d]	1359/8546	0.94	(0.91, 0.98)	0.94	(0.91, 0.98)		
Inclusion of individual lifestyle factors to the basic score							
Basic lifestyle score+sleep	5952/40462	0.77	(0.75, 0.78)	0.77	(0.76, 0.78)		
Basic lifestyle score+physical activity	5952/40462	0.78	(0.77, 0.79)	0.78	(0.77, 0.80)		
Basic lifestyle score+alcohol intake	5952/40462	0.73	(0.72, 0.75)	0.74	(0.72, 0.75)		
Basic lifestyle score+diet quality	1359/8546	0.80	(0.77, 0.83)	0.80	(0.77, 0.83)		
Basic lifestyle score+sleep+physical activity	5952/40462	0.80	(0.80, 0.82)	0.81	(0.79, 0.82)		
Basic lifestyle score+sleep+physical activity+alcohol intake	5952/40462	0.81	(0.80, 0.83)	0.82	(0.80, 0.83)		
Basic lifestyle score+sleep+physical activity+diet quality	1359/8546	0.85	(0.82, 0.87)	0.85	(0.82, 0.87)	0.85	(0.82, 0.87)
Basic lifestyle score+sleep+physical activity+alcohol intake+diet quality	1359/8546	0.87	(0.85, 0.90)	0.87	(0.85, 0.90)		
Level of basic lifestyle score							
Poor (reference)	2970/12248	1.00		1.00		1.00	
Intermediate	1689/10315	0.49	(0.45, 0.53)	0.49	(0.46, 0.54)	0.49	(0.46, 0.54)

Ideal	1293/17899	0.34	(0.31, 0.36)	0.34	(0.32, 0.37)	0.34	(0.32, 0.37)
p trend		*<0.0001*		*<0.0001*		*<0.0001*	
Level of individual lifestyle score factors							
Waist circumference							
Poor (reference)	2522/9292	1.00		1.00			
Intermediate	1940/11714	0.50	(0.46, 0.54)	0.51	(0.47, 0.54)		
Ideal	1490/19456	0.27	(0.25, 0.29)	0.27	(0.25, 0.29)		
p trend		*<0.0001*		*<0.0001*			
Smoking							
Poor (reference)	489/3562						
Intermediate	1626/9376	0.90	(0.81, 1.00)	0.92	(0.83, 1.02)		
Ideal	3837/27524	0.95	(0.89, 1.01)	0.94	(0.88, 1.01)		
p trend		*0.07*		*0.09*			
Total serum cholesterol							
Poor (reference)	1556/6382	1.00		1.00			
Intermediate	2348/14230	0.78	(0.73, 0.83)	0.79	(0.73, 0.84)		
Ideal	2048/19850	0.56	(0.52, 0.61)	0.57	(0.53, 0.62)		
p trend		*<0.0001*		*<0.0001*			
Sleep							
Poor (reference)	628/3949	1.00		1.00			
Intermediate	1861/11604	0.97	(0.91, 1.04)	0.97	(0.91, 1.03)		
Ideal	3463/24909	0.86	(0.78, 0.95)	0.86	(0.78, 0.95)		
p trend		*0.01*		*0.01*			
Physical activity							
Poor (reference)	1927/10862	1.00		1.00			
Intermediate	604/4035	0.89	(0.81, 0.97)	0.89	(0.81, 0.98)		
Ideal	3421/25565	0.77	(0.72, 0.82)	0.77	(0.72, 0.82)		
p trend		*<0.0001*		*<0.0001*			
Alcohol intake							
Poor (reference)	5442/36741	1.00		1.00			
Intermediate	360/2614	0.91	(0.73, 1.12)	0.92	(0.74, 1.14)		
Ideal	150/1107	0.92	(0.77, 1.11)	0.93	(0.77, 1.12)		
p trend		*0.65*		*0.71*			
Diet quality[d]							
Poor (reference)	495/3288	1.00		1.00			
Intermediate	587/3461	0.87	(0.74, 1.02)	0.87	(0.74, 1.02)		
Ideal	277/1797	0.91	(0.77, 1.08)	0.91	(0.77, 1.08)		
p trend		*0.23*		0.24			
Level of combined lifestyle score factors (sleep+physical activity+alcohol intake+diet quality) [d]							

Poor (reference)	575/3200	1.00		1.00	
Intermediate	355/2379	0.98	(0.84, 1.15)	0.98	(0.84, 1.15)
Ideal	429/2967	0.80	(0.70, 0.92)	0.80	(0.69, 0.92)
p trend		*0.42*		*0.38*	
Level of individual lifestyle factors included in the basic score					
Basic lifestyle score+sleep					
Poor (reference)	3767/17200	1.00		1.00	
Intermediate	1261/9565	0.58	(0.53, 0.64)	0.60	(0.54, 0.65)
Ideal	924/13697	0.36	(0.33, 0.39)	0.37	(0.34, 0.40)
p trend		*<0.0001*		*<0.0001*	
Basic lifestyle score+physical activity					
Poor (reference)	2615/10982	1.00		1.00	
Intermediate	2472/16379	0.48	(0.44, 0.52)	0.49	(0.45, 0.53)
Ideal	865/13101	0.31	(0.29, 0.34)	0.32	(0.29, 0.34)
p trend		*<0.0001*		*<0.0001*	
Basic lifestyle score+alcohol intake					
Poor (reference)	2818/11508	1.00		1.00	
Intermediate	2643/19753	0.48	(0.43, 0.53)	0.48	(0.44, 0.54)
Ideal	491/9201	0.27	(0.24, 0.30)	0.28	(0.25, 0.31)
p trend		*<0.0001*		*<0.0001*	
Basic lifestyle score+diet quality					
Poor (reference)	725/3060	1.00		1.00	
Intermediate	294/1955	0.73	(0.62, 0.87)	0.74	(0.62, 0.88)
Ideal	340/3531	0.44	(0.38, 0.51)	0.45	(0.39, 0.52)
p trend		*<0.0001*			
Basic lifestyle score+sleep+physical activity					
Poor (reference)	3261/14779	1.00		1.00	
Intermediate	1178/7610	0.59	(0.55, 0.65)	0.60	(0.55, 0.65)
Ideal	1513/18073	0.42	(0.39, 0.45)	0.43	(0.40, 0.46)
p trend		*<0.0001*		*<0.0001*	
Basic lifestyle score+sleep+physical activity+alcohol intake					
Poor (reference)	3136/13999	1.00		1.00	
Intermediate	2074/15523	0.57	(0.52, 0.63)	0.58	(0.53, 0.63)
Ideal	742/10940	0.35	(0.32, 0.39)	0.36	(0.33, 0.39)
p trend		*<0.0001*		*<0.0001*	
Basic lifestyle score+sleep+physical activity+alcohol intake+diet quality[d]					
Poor (reference)	717/3197	1.00		1.00	
Intermediate	435/3057	0.73	(0.62, 0.86)	0.73	(0.62, 0.87)

Ideal	207/2292	0.49	(0.42, 0.58)	0.49	(0.42, 0.58)		
p trend		*<0.0001*		*<0.0001*			
Basic lifestyle score+sleep+physical activity+diet quality [d]							
Poor (reference)	748/3325	1.00					
Intermediate	434/3093	0.65	(0.54, 0.78)	0.65	(0.54, 0.79)	0.65	(0.54, 0.79)
Ideal	177/2128	0.42	(0.35, 0.50)	0.42	(0.35, 0.50)	0.42	(0.35, 0.50)
p trend		*<0.0001*		*<0.0001*		*<0.0001*	

[a]OR odds ratio, 95% CI confidence intervals. Logistic regression models represent the increase in hypertension per 1-point increase in the lifestyle scores
[b]Model 1 is adjusted for age, sex, and employment country. Model 2 is model 1 adjusted for marital status, education, ethnicity, annual household income, and history of chronic diseases. Model 3 was additionally adjusted for alcohol intake
[c]Hypertension was defined as having SBP≥140mmHg and DBP≥ 90 mmHg, reported diagnosis or on anti-hypertensive medication
[d]Analyzed in a subsample of n=8,546

Table S2. Estimated mean differences in BP associated with a 1 point higher lifestyle scores and their components in a sample of the Airwave Health Monitoring Study (n=40,462) stratified by age [a,b]

	**Systolic blood pressure (mmHg)**<br>Mean difference (95% CI)	**Diastolic blood pressure (mmHg)**<br>Mean difference (95% CI)
**Age ≤ 30 years (n = 6397)**		
Basic lifestyle score (waist circumference+smoking+cholesterol)		
Model 2	-1.58 (-1.80, -1.36)***	-1.69 (-1.86, -1.52) ***
Individual lifestyle risk score factors		
Waist circumference		
Model 2	-3.13 (-3.51, -2.75) ***	-3.20 (-3.48, -2.91) ***
Smoking		
Model 2	-0.09 (-0.50, 0.31)	-0.15 (-0.46, -0.17) **
Total serum cholesterol		
Model 2	-2.18 (-2.64, -1.72)***	-2.49 (-2.85, -2.14)***
Sleep		
Model 2	-0.05 (-0.35, 0.46)	-0.36 (-0.67, -0.05)*
Physical activity		
Model 2	-0.10 (-0.27, 0.05)	-0.83 (-1.09, -0.58)***
Alcohol intake		
Model 2	-0.75 (-1.44, -0.05)*	-0.59 (-1.12, -0.05)*
Diet quality[c]		
Model 2	-0.07 (-0.25, 0.11)	-0.36 (-0.81, 0.09)
Combined lifestyle score factors (sleep+physical activity+alcohol intake+diet quality)[c]		
Model 2	-0.08 (-0.46, 0.31)	-0.52 (-0.81, -0.22)**
Inclusion of individual lifestyle factors to the basic score		
Basic lifestyle score+sleep		

Model 2	-1.14 (-1.33, -0.95)***	-1.31 (-1.46, -1.17)***
Basic lifestyle score+sleep+physical activity		
Model 2	-0.77 (-0.93, -0.62)***	-1.10 (-1.22, -0.98)***
Basic lifestyle score+sleep+physical activity+alcohol intake		
Model 2	-0.78 (-0.94, -0.63)***	-1.09 (-1.21, -0.97)***
Basic lifestyle score+sleep+physical activity+alcohol intake+diet quality[c]		
Model 2	-0.49 (-0.78, -0.20)**	-0.81 (-1.03, -0.59)***
**Age 30 to ≤ 40 years (n = 13,701)**		
Basic lifestyle score (waist circumference+smoking+cholesterol)		
Model 2	-1.81 (-1.96, -1.66)***	-2.00 (-2.07, -1.85)***
Individual lifestyle risk score factors		
Waist circumference		
Model 2	-3.36 (-3.61, -3.10) ***	-3.52 (-3.72, -3.33)***
Smoking		
Model 2	-0.11 (-0.43, 0.19)	-0.26 (-0.50, -0.03)*
Total serum cholesterol		
Model 2	-2.29 (-2.59, -2.00)***	-2.51 (-2.73, -2.28)***
Sleep		
Model 2	-0.19 (-0.50, 0.12)	-0.42 (-0.65, -0.19)**
Physical activity		
Model 2	-0.04 (-0.20, 0.27)	-1.00 (-1.16, -0.81)***
Alcohol intake		
Model 2	-1.00 (-1.46, -0.44)**	-0.52 (-0.91, -0.13)**
Diet quality[d]		
Model 2	-0.05 (-0.21, 0.31)	-0.56 (-0.81, -0.23)**
Combined lifestyle score factors (sleep+physical activity+alcohol intake+diet quality) [d]		
Model 2	-0.27 (-0.55, -0.10)*	-0.71 (-0.92, -0.50)**
Inclusion of individual lifestyle factors to the basic score		
Basic lifestyle score+sleep		
Model 2	-1.41 (-1.55, -1.28)***	-1.58 (-1.68, -1.48)***
Basic lifestyle score+sleep+physical activity		
Model 2	-0.96 (-1.07, -0.86)***	-1.30 (-1.38, -1.22)***
Basic lifestyle score+sleep+physical activity+alcohol intake		
Model 2	-0.97 (-0.94, -0.63)***	-1.28 (-1.36, -1.20)***
Basic lifestyle score+sleep+physical activity+alcohol intake+diet quality[d]		
Model 2	-0.72 (-0.92, -0.51)**	-1.00 (-1.15, -0.85)***
**Age 40 to ≤ 50 years (n = 15,338)**		
Basic lifestyle score (waist circumference+smoking+cholesterol)		
Model 2	-2.46 (-2.62, -2.29)***	-2.25 (-2.36, -2.14)***
Individual lifestyle risk score factors		
Waist circumference		

Model 2	-4.14 (-4.41, -3.87)***	-3.90 (-4.09, -3.72)***
Smoking		
Model 2	-0.23 (-0.60, 0.12)	-0.46 (-0.70, -0.21)**
Total serum cholesterol		
Model 2	-2.29 (-2.59, -2.00)***	-2.53 (-2.73, -2.32)***
Sleep		
Model 2	-0.19 (-0.52, 0.15)	-0.51 (-0.75, -0.28)***
Physical activity		
Model 2	-0.39 (-0.64, -0.13)**	-1.16 (-1.33, -0.98)***
Alcohol intake		
Model 2	-0.59 (-1.18, -0.05)*	-0.73 (-1.14, -0.32)**
Diet quality [e]		
Model 2	-0.11 (-0.33, 0.31)	-0.42 (-0.71, -0.12)**
Combined lifestyle score factors (sleep+physical activity+alcohol intake+diet quality) [e]		
Model 2	-0.16 (-0.44, 0.13)	-0.65 (-0.84, -0.45)***
Inclusion of individual lifestyle factors to the basic score		
Basic lifestyle score+sleep		
Model 2	-1.92 (-2.07, -1.78)***	-1.83 (-1.93, -1.73)***
Basic lifestyle score+sleep+physical activity		
Model 2	-1.38 (-1.50, -1.26)***	-1.49 (-1.57, -1.41)***
Basic lifestyle score+sleep+physical activity+alcohol intake		
Model 2	-1.34 (-1.46, -1.23)***	-1.45 (-1.53, -1.37)***
Basic lifestyle score+sleep+physical activity+alcohol intake+diet quality [e]		
Model 2	-0.90 (-1.11, -0.69)***	-1.06 (-1.20, -0.92)***
**Age > 50 years (n = 5,026)**		
Basic lifestyle score (waist circumference+smoking+cholesterol)		
Model 2	-2.34 (-2.70, -1.98)***	-1.72 (-1.93, -1.52)***
Individual lifestyle risk score factors		
Waist circumference		
Model 2	-3.90 (-4.47, -3.33)***	-3.14 (-3.46, -2.81)***
Smoking		
Model 2	-0.21 (-0.56, 0.98)	-0.27 (-0.17, 0.72)
Total serum cholesterol		
Model 2	-2.52 (-3.15, -1.90)***	-1.63 (-1.99, -1.26)***
Sleep		
Model 2	-0.23 (-0.46, 0.92)	-0.15 (-0.55, 0.25)
Physical activity		
Model 2	-0.31 (-0.82, 0.21)	-0.59 (-0.89, -0.29)***
Alcohol intake		
Model 2	-0.11 (-1.03, 1.24)	-0.24 (-0.90, 0.42)

Diet quality[f]		
Model 2	-0.40 (-1.25, 0.45)	-0.57 (-1.05, -0.08)*
Combined lifestyle score factors (sleep+physical activity+alcohol intake+diet quality) [f]		
Model 2	-0.22 (-0.80, 0.37)	-0.42 (-0.75, -0.09)*
Inclusion of individual lifestyle factors to the basic score		
Basic lifestyle score+sleep		
Model 2	-1.73 (-2.04, -1.42)***	-1.34 (-1.52, -1.16)***
Basic lifestyle score+sleep+physical activity		
Model 2	-1.26 (-1.52, -1.00)***	-1.07 (-1.22, -0.92)***
Basic lifestyle score+sleep+physical activity+alcohol intake		
Model 2	-1.18 (-1.44, -0.93)***	-1.02 (-1.16, -0.87)***
Basic lifestyle score+sleep+physical activity+alcohol intake+diet quality[f]		
Model 2	-1.00 (-1.43, -0.58)***	-0.79 (-1.03, -0.55)***

[a]Values are presented as mean (95% CI, confidence intervals); *P-value<0.05, **P-value<0.001 ***P-value<0.0001

[b]Model 1 is adjusted for sex, and employment country. Model 2 is model 1 adjusted for marital status, education, ethnicity, annual household income, and history of chronic diseases

[c]Analysed in a subsample of n=1,341

[d]Analysed in a subsample of n=2,615

[e]Analysed in a subsample of n=3,396

[f] Analysed in a subsample of n=1,194

Table S3. Estimated mean differences in BP associated with a 1-point higher lifestyle scores in subcohorts of the Airwave health monitoring study [a,b]

	**Systolic blood pressure (mmHg)** Mean difference (95% CI)	**Diastolic blood pressure (mmHg)** Mean difference (95% CI)
Participants with a self-reported diagnosis of hypertension, users of antihypertensive drugs, and those with prevalent cardiovascular diseases and diabetes [c]		
Model 2	-0.93 (-1.22, -0.64)***	-1.01 (-1.18, -0.84)***
Excluding energy misreporters [d]		
Model 2	-2.04 (-2.29, -1.80)***	-1.84 (-2.00, -1.68)***

[a] Values are presented as mean (95% CI, confidence intervals); ***P-value<0.0001

[b] Model 2 is adjusted for age, sex, employment country, marital status, education, ethnicity, annual household income, history of chronic diseases

[c] Subcohort of participants with a self-reported diagnosis of hypertension, users of antihypertensive drugs, and those with prevalent cardiovascular diseases and diabetes, n=5,840

[d] Subcohort excluding energy under-reporters, n=6,904
